# Ecosystem services classification: A systems ecology perspective of the cascade framework

**DOI:** 10.1016/j.ecolind.2016.11.030

**Published:** 2017-03

**Authors:** Alessandra La Notte, Dalia D’Amato, Hanna Mäkinen, Maria Luisa Paracchini, Camino Liquete, Benis Egoh, Davide Geneletti, Neville D. Crossman

**Affiliations:** a European Commission - Joint Research Centre, Directorate D – Sustainable Resources, Via Enrico Fermi 2749, 21027 Ispra, VA, Italy; b University of Helsinki, Department of Forest Sciences, Latokartanonkaari 7, Helsinki, 00014, Finland; c Lappeenranta University of Technology, School of Energy Systems, Sustainability Science, Saimaankatu 11, 15140 Lahti, Finland; d Council for Scientific and Industrial Research, Natural Resources and The Environment, PO Box 320, Stellenbosch 7599, South Africa; e School of Agricultural, Earth and Environmental Sciences, University of KwaZulu-Natal, 27 Private Bag X01, Scottsville 3209, South Africa; f University of Trento, Department of Civil, Environmental and Mechanical Engineering, Via Mesiano 77, 38123 Trento, Italy; g CSIRO Land and Water, Waite Campus, Adelaide, South Australia, 5064, Australia

**Keywords:** Systems ecology, Ecosystem functioning, Cascade framework, Ecological theory, Ecosystem service classification

## Abstract

Ecosystem services research faces several challenges stemming from the plurality of interpretations of classifications and terminologies. In this paper we identify two main challenges with current ecosystem services classification systems: i) the inconsistency across concepts, terminology and definitions, and; ii) the mix up of processes and end-state benefits, or flows and assets. Although different ecosystem service definitions and interpretations can be valuable for enriching the research landscape, it is necessary to address the existing ambiguity to improve comparability among ecosystem-service-based approaches. Using the cascade framework as a reference, and Systems Ecology as a theoretical underpinning, we aim to address the ambiguity across typologies. The cascade framework links ecological processes with elements of human well-being following a pattern similar to a production chain. Systems Ecology is a long-established discipline which provides insight into complex relationships between people and the environment. We present a refreshed conceptualization of ecosystem services which can support ecosystem service assessment techniques and measurement. We combine the notions of biomass, information and interaction from system ecology, with the ecosystem services conceptualization to improve definitions and clarify terminology. We argue that ecosystem services should be defined as the interactions (i.e. processes) of the ecosystem that produce a change in human well-being, while ecosystem components or goods, i.e. countable as biomass units, are only proxies in the assessment of such changes. Furthermore, Systems Ecology can support a re-interpretation of the ecosystem services conceptualization and related applied research, where more emphasis is needed on the underpinning complexity of the ecological system.

## Highlights

• Different ecosystem service definitions and interpretations create too much ambiguity.
• The cascade model is used as framework, and Systems Ecology as theoretical basis.
• The notions of biomass information and interaction enrich a refreshed conceptualization.
• The cascade framework shifts from a ‘two dimensional’ to a ‘telescopic’ perspective.
• This perspective can emphasize the ecological dimension and its complex reality.

## 1 Introduction

Ecosystem services is now widely used among scientists and policy makers to highlight the importance of the environment (including biodiversity) in sustaining human livelihoods (Convention on Biological Diversity, 2010, Convention on Biological Diversity, 1998, Costanza and Kubiszewski, 2012, Maes et al., 2016). An important milestone of ecosystem service research was the Millennium Ecosystem Assessment (MA, 2005) which made prominent the idea that human well-being depends on ecosystems, and that such linkages can be tracked and framed through the notion of ecosystem services. The MA found that more than 60% of ecosystem services is being degraded or transformed endangering future human well-being.

Ecosystem services research has since progressed at different levels—from theoretical conceptualization to practical applications (see Braat and de Groot, 2012, Egoh et al., 2012, Seppelt et al., 2011, Potschin et al., 2016 for a review). This work has been supported by several international initiatives such as The Economics of Ecosystem and Biodiversity (TEEB, 2010), the UK National Ecosystem Assessment (UK NEA, 2011) and several European Union research projects.^1^ In addition, some organizations have supported this process with modeling tools such as the US Natural Capital Project with the Integrated Valuation of Ecosystem Services and Tradeoffs (InVEST) tool. The private sector have also adopted the concept through initiatives such as the Natural Capital Coalition (NCC), the World Bank’s Wealth Accounting and the Valuation of Ecosystem Services (WAVES), the accounting system developed by the London Group, which is also being adopted by the United Nations Environmental Program (UNEP).

However, there has been inconsistency in developing a framework within which such research and policy assessments are carried out. The he MA (2005) and subsequent ecosystem services literature (Boyd and Banzhaf, 2007, Fisher et al., 2009, Landers and Nahlik, 2013, Staub et al., 2011, Wallace, 2007) have developed many different conceptual and empirical frameworks and assessment of changes in ecosystems, their consequences for humans, and actions for sustainable use of these ecosystems (Albert et al., 2015). The existence of numerous ecosystem service conceptualizations and classification systems has led to a plurality in the interpretation of ecosystem services and related terminology and definitions when it comes to applications (Boerema et al., 2016). Large differences in interpretation are found in the meaning of biophysical structure, ecological functions, intermediate services and final services (e.g. Landers and Nahlik, 2013, Mononen et al., 2016; Spangenberg et al., 2014; UK NEA, 2011, TEEB, 2010). The consequence of such differences is the ecosystem service classification systems have poor correspondence of services with benefits and blurred distinctions between intermediate and final services. Among these, the Common International Classification for Ecosystem Services (CICES), proposed by the European Environment Agency, has become an important frame of reference for ecosystem services research (Maes et al., 2014). CICES and most ecosystem services literature are based on and influenced by the cascade framework proposed by Haines-Young & Potschin in 2010 (Haines-Young and Potschin, 2010, Potschin and Haines-Young, 2016). The purpose of the cascade framework is in fact to show the pathway of ecosystem services from ecological structures and processes to human well-being.

In this context, the need to develop a framework to assess ecosystem services is a priority in ecosystem services research. Although individual interpretations enrich the research landscape, the ambiguity must be addressed so that a more rigorous framework for ecosystem services can be developed and adopted. Such a framework would improve comparability among ecosystem-service-based approaches and would provide a standardized approach for ecosystem assessments at global and national scales. The further evolution of ecosystem services concepts and frameworks could draw from the field of systems ecology which can provide insights into our understanding of the different aspects of ecosystem functioning that contributes to ecosystem services. This interdisciplinary field of systems ecology adopts a holistic approach to the study of ecological and human systems. Concepts from ecological theory have been already discussed in previous literature in relation to ecosystem services, e.g. ecological integrity and complexity, resilience (Kremen, 2005, Brand, 2008). Our paper aims to systematically adopt key concepts from systems ecology to re-define ecosystem services and the related cascade framework. The contribution of our paper is to present a refreshed conceptualization of ecosystem services through the lens of systems ecology.

We firstly identify the main challenges associated with the various interpretations of the cascade framework (Section 2.1) and of the existing classification systems whose structure and meaning does depend on the chosen theoretical framework (Section 2.2). Secondly, we introduce key concepts from the discipline of systems ecology (Section 3) to address the identified challenges (Section 4). We finally conclude by discussing the contribution of our refreshed conceptualization of ecosystem services (Section 5).

## 2 Current challenges in ecosystem services research

### 2.1 Challenges with the use of the ecosystem services cascade

The cascade framework proposed by Haines-Young and Potschin (2010) links natural systems to elements of human well-being, following a pattern similar to a production chain: from ecological structures and processes generated by ecosystems, to the services and benefits eventually derived by humans. The advantage of this framework is to effectively communicate societal dependence on ecosystems.

Challenges arise when applying this cascade framework in practice, due to the simultaneous presence in the framework of bio-centered and human-centered spheres. This means that ecosystem services assessments include:

• observations from a bio-centred or holistic approach- i.e. biophysical structures and processes/functions belonging to the ecological sphere and which are considered as a whole,
• observations from a reductionist or human-centred approach- i.e. ecosystem services which are projected towards the human end-use side individually.

This challenge is evident when we try to measure ecosystem services, which are categorized and accounted for individually.^2^

In addition, different definitions of ecosystem services and in particular of the elements in the cascade framework are found in the literature: biophysical structure, process, function, service, benefit.^3^ As an example, Table 1 summarizes the definitions provided in recent ecosystem services studies. For instance, ecosystem structure is often poorly distinguished from processes. Wallace (2007, p. 237) proposes that ‘an important distinction [between the two] is that the former are generally tangible entities described in terms of amount, while the latter are […] generally described in terms of rates’.

Furthermore, the word function is generally used interchangeably with ecological process and/or ecosystem service. According to Jax (2005), the term ‘function’ is often used too ambiguously. Ecosystem services are generally defined as the ecosystem processes considered useful to humans (MA, 2005, TEEB, 2010). In the same light, some studies (ref. Table 1) that have assessed, mapped or valued ecosystem services, use services and benefits as synonyms. Benefits are in some cases considered as tangible natural resources derived from provisioning services (e.g. crops, wood, water), or some regulating services (e.g. clean water for multiple uses provided by water purification). Benefits, however, can also be intangible (e.g. recreation opportunities offered by nature). Haines-Young and Potschin (2009, p. 17) propose a ‘pragmatic way forward’, stating that ‘the main issue is to ensure the rigor of the outputs from our analysis and not become preoccupied with definitions, hence efforts should be directed to: achieving consistent valuation and no double counting’.

More unified and shared definitions, however, can be helpful in ensuring the rigor of practical assessments, and allow a degree of comparability among studies. In particular, it is important to distinguish between service, process, and benefit. Braat and de Groot (2012) argued that ecosystem services contain ‘the product component (traditionally called “goods”)’, but they suggest that ‘in the next stage of development of the concept, the distinction between goods and services should be re-established’. When referring to the cascade framework, the terminology includes benefits rather than goods. The challenge of separating services from goods and/or benefits is further explored in the next section.

### 2.2 Challenges in the current ecosystem services classifications

Any application of an ecosystem service-based approach starts with choosing the services to be assessed (and valued) from a list of services, i.e a classification system. Classification systems are usually based on a theoretical framework whose principles and concepts are reflected in the meaning and structure of the items presented. It is thus important to explore the main classification systems, in order to highlight the embedded notions they state. For example, the Millennium Ecosystem Assessment (2005) was the first to attempt to group ecosystem services into four categories: provisioning services (e.g. food, fibers, fuel, genetic resources); regulating services (e.g., water purification and regulation, climate regulation, extreme events and disease mitigation); supporting services (e.g., primary production and nutrient cycling); and cultural services (e.g., eco-tourism and recreation, aesthetic and spiritual values). This categorization provided a sound basis to launch ecosystem services research and applications, but it does not constitute a proper taxonomy. In the cascade framework (Haines-Young and Potschin, 2012), supporting services are considered a ‘function’ rather than a ‘service’. Following the MA, the TEEB classification (2010) also explicitly referred to the cascade framework but refined the distinction between services and benefits. The idea of supporting services in TEEB was not further developed. Instead a new ‘habitat services’ group was introduced, including ‘maintenance of life cycles’ and ‘maintenance of genetic diversity’

Since some ecosystem service categories overlap, there is a risk of double counting in valuation, which therefore requires clear separation between intermediate and final service. The US Environmental Protection Agency has proposed additional classifications to avoid double counting. These include Final Ecosystem Goods and Services Classification System (FEGS-CS) (Landers and Nahlik, 2013) and the National Ecosystem Services Classification System (NESCS) (Rhodes, 2015). In both classification systems the main focus is on benefits and beneficiaries. This is in line with the study by Boyd and Banzhaf (2007) that suggest to account for ‘components of nature directly enjoyed, consumed or used to yield human well-being’. FEGS-CS classification proposes two criteria to define goods and services: i) the potential good or service is valued by a beneficiary, and; ii) the potential good or service is connected to at least the hydrosphere and lithosphere. In FEGS-CS processes such as photosynthesis or carbon sequestration are labeled all together as ‘ecosystem structural components’ and considered as intermediate goods and services. These are excluded because they are not directly used by humans. Similarly, NESCS classification represents distinct pathways through which final ecosystem services enter human systems. This classification approach focuses on end categories of uses and users, and is aligned with the North America national accounts classification system. NESCS emphasizes the connection between the ‘end-product of nature’ and the human ‘direct uses’ as tangible and intangible benefits.

CICES is one of the most popular classifications currently and is being used by scientists and policy makers around the globe but particularly from Europe. Similar to the TEEB classification, CICES does not include the MA (2005) ‘supporting services’, but merges the TEEB (2010) ‘habitat services’ with regulating services, in a category called ‘regulating and maintenance services’. Compared to FEGS-CS and NESCS, CICES does promote a clear distinction between ecosystem services and ecosystem benefits. In the latest version of the cascade framework that underpins CICES (Potschin and Haines-Young, 2016), ecosystem services are explicitly indicated as final services, while biophysical structure and function are indicated as supporting or intermediate services. Final ecosystem services are the contributions that ecosystems make to human well-being as flows. Ecosystem goods and benefits are created or derived by people from final ecosystem services.

**Fig. 1:**
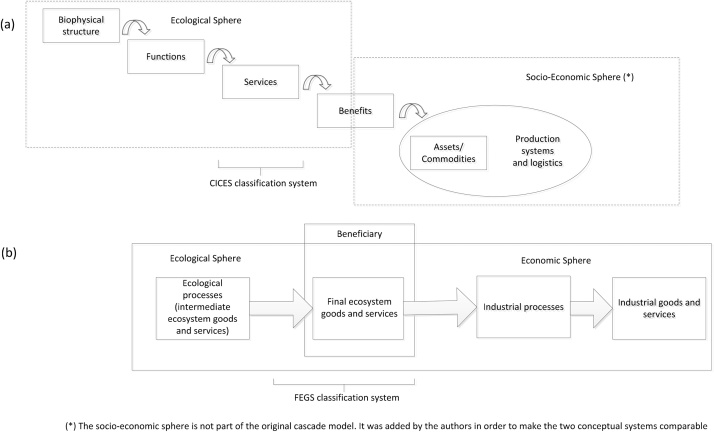
A comparison of CICES and FEGS classifications.

The differences between FEGS-CS and CICES are subtle and are explained with the assistance of Fig. 1: a) the cascade framework that constitutes the theoretical background of CICES, and; b) the conceptual framework of the FEGS-CS. FEGS-CS places emphasis on the benefits, beneficiaries and the socio-economic system, while CICES places greater emphasis on the ecological system. In fact we need to add an additional box (i.e. assets/commodities) in the cascade framework to have a more consistent view of the two models. In this additional box the benefits enter into a production process that makes it a marketable good, an economic asset, a commodity. Although ecosystem services are identified considering human needs and demand, we choose in Fig. 1a to have the socio-economic systems starting at the ‘benefit’ box because at this stage the real use can take place and because this is the only way to consistently compare the two theoretical frameworks. By comparing these two classifications to each other and to the cascade framework, we observe that FEGS-CS classification regards different benefits rather than ecosystem services.

The most appropriate classification system should be chosen based on its fit-for-purpose (Heink et al., 2015, Spangenberg and Settele, 2010), i.e. whether the ecosystem service analysis intends to focus more on ecological systems (e.g. considering impacts on and pressures from the socio-economic side) or on socio-economic systems (e.g. the benefits derived by society). It is however important to be aware of the existing limitations of each classification system.

## 3 The nature of ecosystem services: a systems ecology perspective

**Fig. 2:**
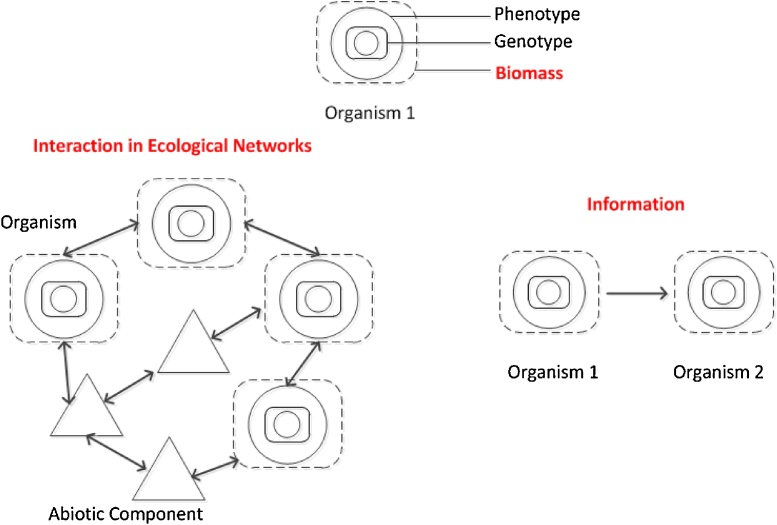
A schematic representation of biomass, information, interaction.

In the theory of systems ecology, Jørgensen (2012) proposed three fundamental notions as the basis of ecological systems: 1) biomass, 2) interaction and 3) information in ecological networks. In this section we argue that ecosystem services have in fact been conceptualized as either (bio)mass, information or interaction (Fig. 2). We adopt the following definitions of these key concepts.

**Biomass** is biological material derived from living or dead organisms. The quality aspect of biomass is also relevant, e.g. based on protein synthesis and evolution.

**Interaction** occurs in a network as components have an effect upon one another. Interactions are therefore the relationships between and among biotic and abiotic components, sometimes characterized by a temporal pattern; such relationships can be bi- or multi-directional, as opposed to the unidirectional causal effect of information. In ecological networks, interactions might result in emergent properties of the system. Emerging properties in a system cannot be predicted or explained by the sum of the components alone, because the latter do not exhibit such properties themselves (Edson et al., 1981, Odum, 1977). Social behaviour in animals is an example, such as ‘the ability of large populations of simple, identical units (for example, spin magnets) to self-organize, form patterns, store information, and reach “collective decisions” (Parrish and Edelstein-Keshet, 1999). Interactions in an ecological network can also be defined as **ecological processes**.

**Information** can be considered a sub-category of interaction; information is “conveyed or represented by a particular arrangement or sequence of things, including for example, genetically transmitted information” (Oxford Dictionary Online, 2014). Information can influence (intentionally or not) the formation or transformation of other patterns. Organisms interact with their environment not just by exchanging material and energy as traditionally viewed in Ecology, but also by exchanging information (Dusenbery, 1992). The process of acquiring information involves a mechanistic phase of information capture by a receptor, such as a sensory organ, and a functional phase of information de-codification. This is the ability to recognize and process that information as ‘knowledge’ (Guilford and Dawkins, 1991). Consequently, exchange of information occurs between two (or more) organisms when the ‘receiver’ organism(s) is able to capture and process the information of the ‘sender’. While information plays a role in the generation of all ecosystem services (e.g. genetic information), in this article we specifically define information as the one humans receive and process.

An organism expresses and conveys biomass, information and interactions via its genotype and/or phenotype (Fig. 2). We refer here to the extended phenotype (Dawkins, 1982), which includes the appearance of an organism (morphology, development, biochemical and physiological processes, etc.) as well as properties external to the body (phenology, behaviour, products of behaviour). For example, the silk produced by the silkworm (*Bombyx mori*) is essentially biomass, derived from its chrysalis during the metamorphosis. Therefore, the ecosystem service (in this case the silk produced by the silkworm) is not a direct product of its body mass, but rather an expression of its phenotype.

Based on the given definitions of biomass, information and interaction, we can examine the current classification of ecosystem services. Most provisioning services are conceptualized as (bio)mass e.g. food, fiber, water (de Groot et al., 2002; MA, 2005, TEEB, 2010). Genetic resources represent an exception among provisioning services, since we consider them as information. In fact, the genotype or phenotype of an organism can contribute to develop drugs or to bioengineering. Regulating services are based on interactions among biotic and abiotic elements of the ecosystems: for example water purification derives from the overall mechanical and chemical capacity of abiotic soil, soil biota and vegetation to trap and ‘convert’ sediments, nutrients, pollutants or pathogens. Cultural services derive from information. For example, we are able to receive the information from an amenity landscape given the human ability to perceive (receptor) and appreciate beauty (decodification and interpretation). This information might influence humans, for example triggering inspiration, a physiological relaxation, a sense of fulfilment, or a spiritual experience.

**Fig. 3:**
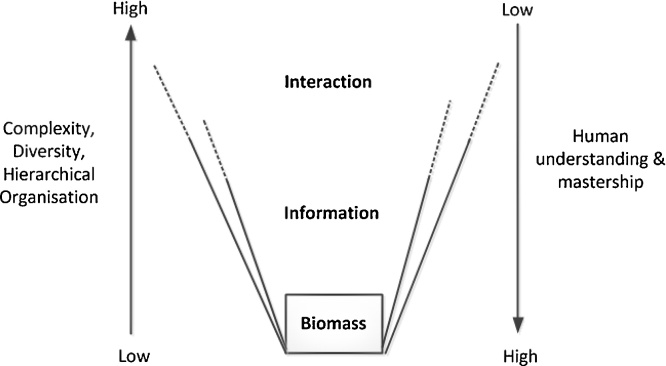
The nature of biomass, information and interaction in Systems Ecology, and the human understanding and mastership of these concepts.

Drawing from thermo-dynamics, Jørgensen (2012, chapter 13) proposes the following ideas: growth of matter is limited by energy input and availability of inorganic elements. The growth of information and interactions in networks is driven by evolution (thus linked to diversity) and has potential to expand (Faith et al., 2010) (Fig. 3): information and interactions have overall increased in the history of living organisms. Unlike matter and energy, information and interactions can disappear without trace when the material support (biomass) is destroyed.^4^ Thus, biomass, information and interactions are characterized by increasing complexity and operate at different hierarchical levels. Biodiversity is at the basis of this complexity: the more diversity, the more information and interactions. The very definition of Biodiversity (Convention on Biological Diversity, 1992) refers to the hierarchical organization of all organisms as well as the functional characteristics of each level. The processes at one level of organization determine the conditions in the next level, while higher levels regulate and control lower levels by feedback. For example, species diversity influences ecosystem properties and functioning, and *vice versa*. It has to be noted that this is an artificial categorization, since in nature the hierarchy is not clearly defined, but more fluid.

## 4 Refreshing the conceptual approach to ecosystem services

### 4.1 Re-defining the cascade framework based on system ecology

Based on the definitions above, we address the challenges in ecosystem services research identified in section 2. We combine the notions of biomass, information and interaction with ecosystem services conceptualization to improve definitions and clarify terminology. We recall Palmer and Febria (2012) to show the linkages through the cascade chain: the components of an ecosystem (that represent the structure) interact with dynamic biophysical processes (that are functions) to produce goods and services that people rely on. We argue that ecosystem services should exclusively be considered as the interactions of the ecosystems that produce a change in human well-being (Table 2). We therefore propose that ecosystem services are not individual ecosystem components or goods. In addition, while all ecosystem services are derived from ecological processes (or socio-ecological processes Mononen et al., 2016) not all processes produce ecosystem services. Some processes may not be of use to humans, but this does not negate their importance. Ecosystem function and ecological processes are considered here as synonyms.

**Fig. 4:**
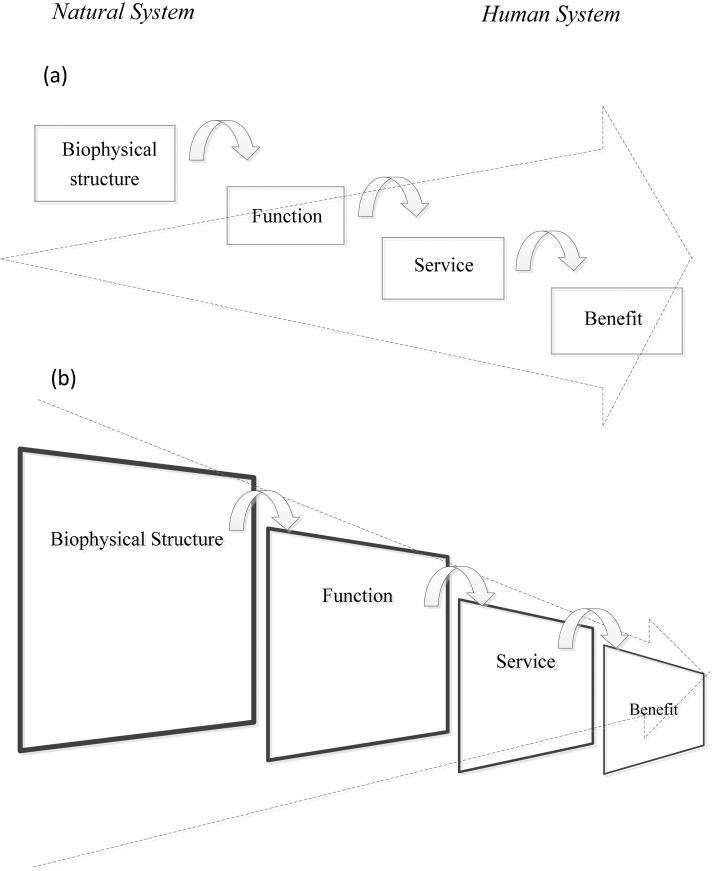
From a 2D to a telescopic cascade framework (a) Traditional understanding of the cascade framework with emphasis on end-use benefits; (b) Systems Ecology re-interpretation of the cascade framework, with emphasis on the underpinning complexity of the ecological system.

Due to the utilitarian nature of ecosystem services, research and policy tend to emphasize end-use benefits rather than the underpinning ecosystem structures and processes (see ‘Traditional understanding of the cascade framework’ in Fig. 4). We propose a modified cascade framework to shift perspective toward ecosystems (see ‘systems ecology re-interpretation of the cascade framework’ in Fig. 4). In Fig. 4 we represent the flow from an ecological perspective. The elements of the cascade are not ‘equal’. It is thus not enough to establish a causal sequence among the elements of the cascade because the inherent complexity of each stage must be highlighted.

To acknowledge this complexity, the hierarchical organization is a crucial concept in systems ecology. Hierarchical levels include atoms, cells, organs, species, populations, ecosystems, landscape, regions and the ecosphere (Jørgensen, 2012). Each level integrates the functions of the lower level.^5^ When we consider the hierarchy from a vertical perspective, each level is constrained from the upper level and from the lower level. However, there is also a horizontal perspective. There is cooperation among the components, which creates networks, where interactions take place.

In many representations of the cascade framework natural capital is considered as examples of benefits (reported as assets or commodities depending on the degree of human intervention in the production process). Natural capital, such as fiber and food, are biomass. From a vertical (hierarchical) perspective these components represent a lower level, while populations of organisms are a higher level. Populations in turn represents a lower level compared to the ecosystem. Different levels interact between each other vertically. In addition, interactions among biotic and abiotic components exist also at horizontal level. Vertical and horizontal interactions constitute the service.

Based on the hierarchical organization drawn from systems ecology, it is possible to highlight the difference between service and benefits. A service is a process and is determined by the horizontal and vertical networking activity. Benefits are individual components, countable as a biomass unit, and a vehicle for ecosystem service enjoyment (Matthies et al., 2016). In the current cascade framework, great emphasis is converging on the benefit, because this is most relevant to humans. It is not our intention to downplay the importance of benefits (and thus the ‘humans’ role in co-producing ecosystem services). We, however, argue for a shift of perspective from a ‘two dimensional’ to a ‘telescopic’ cascade framework which emphasizes the ecological dimensions and complex reality.

The implications of a hierarchical organization are in line with the understanding of ecosystems at the basis of the cascade framework: upper levels change more slowly than lower levels. Variations and disturbances of upper levels may affect the lower levels; the other way round, however, is less frequent, because lower level disturbances are mitigated at upper level (Jørgensen, 2012).^6^ For example, assuming an initial healthy state of the ecosystem, when a single component of the population is removed (e.g. a tree from a forest or one animal from a population), the regeneration capacity is not affected, the functioning of the ecosystem is maintained at a healthy state. When a clear-cut takes place or the species become rare or extinct, then the entire habitat will be affected (e.g. the forest will not be there anymore and the food chain will change).

Any assessment and valuation intended to provide a sustainable policy for the medium and long term cannot ignore the ecological system side of the cascade. The existence of the social system is guaranteed by the proper functioning of the ecological system. The value of the ecological system is intrinsic, and the approach is holistic, bio-centric and positivist. The ecosystem services narrative is part of the human system whose value is utilitarian, and its approach reductionist and human-centered.

### 4.2 Comparing the renewed definition of ecosystem services to CICES classification

We proceed by comparing the concepts introduced from system ecology to the CICES classification and the cascade framework. In Table 3 we list the correspondence between CICES classes and our terminology. This analysis does not intend to add a new level of complication to the ecosystem services conceptualization. Rather it aims at clarifying the difference between ecosystem services and benefits and to improve consistency in the classification of ecosystem services.

Among the list of ecosystem services proposed by CICES, some of them do not meet the requirements for our definition of ecosystem services (i.e. processes) (Table 3). For example, all CICES provisioning services are benefits (i.e. biomass). Provisioning services include for example cultivated crops. However, the ecosystem service is in fact the process to generate crops and plants, rather than the crops and plants themselves. The use of the benefit as a proxy for the service is a common practice, but it might result in double counting. Thus, the resulting benefit from e.g. regulating services should be articulated clearly, so that overlaps with provisioning services are known. For example, benefits from pollination may overlap with cultivated crops; water flow maintenance may overlap with water supplied; or maintaining nursery populations and habitats may overlap with food (fish) provisioning (Liquete et al., 2016a). When performing the trade-off assessment, we do not suggest ignoring regulating services, but rather to carefully consider between provisioning and regulating services.

In CICES the list of services (in particular regulating services) sometimes includes functions and biophysical structures. For instance, ‘chemical condition’ is a property or component of the system and not a process. It is thus part of the biophysical structure. The ecological interactions among components, such as ‘hydrological cycle’ and ‘ventilation and transpiration’ are processes that take place within the ecosystem, and not the flow of an individual service that produces a direct change in human well-being. Differently from benefits, the biophysical structure cannot be a proxy for the service^7^: they are what allows the service flow to be generated (cf. Mononen et al., 2016). In CICES existence and bequest values are listed as services: when attempting a monetary valuation, existence and bequest non-use values are concepts that facilitate the choice of the valuation technique to be adopted, but they are not themselves ecosystem services. Systems ecology theory can thus provide guidance for ecosystem service assessments: Table 3 presents a new classification approach for ecosystem services assessments. In Table 3 we attempt to track correspondence with the different typologies of modeling techniques. By referring to the systems ecology categories of biomass, interaction and information we could state how complex the level of modeling should be.

When ecosystem services are identified as biomass, measurement will require the collection of environmental statistics and inventories. This is the case for many provisioning services, where data is usually extracted from agriculture and forestry statistical databases and inventories, or from market transactions, rather than biophysical processes. Simple and available indicators can be used, such as land-use and land-cover data, biodiversity monitoring maps, or national forest inventories. In this case, rather than assessing the service itself, the benefit is used as proxy for the ecosystem service. This is most relevant to provisioning services and the current practice of assessment.

When ecosystem services are identified as interaction, then ecological modeling or monitoring is needed. To correctly assess the service, the nature of the process should be understood, described analytically and measured. This is the case for some regulating services (i.e. all those services that directly involve biogeochemical cycles) where process-based modeling would better fit the purpose, because the model should be able to represent/replicate the ecosystem functioning (e.g. Liquete et al., 2016b). There are, however, cases in which spatial modeling and statistical modeling could serve the assessment purpose. In spatial modeling algorithms based on spatial features are used and/or different indicators are linked with land use data to derive more complex indicators (see for example Zulian et al., 2013). In statistical models ecosystem services are estimated based on known explanatory variables such as soils, climate, etc., using a statistical relation. This can be the case for those services in which the morphological features do play an important role (e.g. storm and flood protection, soil erosion protection) or the presence of species determines the ‘amount’ of the service (e.g. maintaining nursery populations and habitats).

When the ecosystem services are identified as information, the assessment technique might require calculation of spatially explicit, complex indicators. Information does not require bio-physical modeling, but spatial modeling could be used. All cultural services involve information, and they are generally assessed through questionnaires and mental models. In some cases (e.g. outdoor recreation) the spatial component could play an important role in terms of distance; in other cases it may just be a matter of linking different indicators to make them spatially explicit.

## 5 Discussion and conclusion: advantages of applying the revised conceptual framework

The timing in clarifying and operationalize ecosystem services classification and measurements has never been more critical. As ecosystem services become integrated into policy instruments, the need to standardize definitions is essential for monitoring and comparing policy outcomes following different scales of investment (Bennett et al., 2015, Guerry et al., 2015). Our intention in this article is to provide some clarity to address issues related to ecosystem services definition and conceptualization highlighted by others (Fisher and Turner, 2008, Fisher et al., 2009, Wallace, 2007). Drawing from systems ecology, we adopt the key concepts of biomass, interaction and information, including the idea of different levels of complexity among these (Jørgensen, 2012). In this study, we have used our understanding from systems ecology to apply it to the ecosystem services conceptualization. We believe that the concepts from system ecology can support a more consistent definition of ecosystem services and other elements of the cascade framework developed by Haines-Young and Potschin (2010).

The cascade framework and related ecosystem services definition is often approached with an emphasis on services and benefits. Several authors have identified the need to delineate between direct and indirect ecosystem services (*intermediate* and *final)* for the sake of economic valuation and natural capital accounting, where only benefits from final services can be aggregated (e.g. Fisher et al., 2009, Heink et al., 2015). This approach mitigates the risk of double counting, but it might be overly reductionist. Furthermore, distinctions between ecosystem capacity and actual supply or use of the ecosystem services has been proposed (Albert et al., 2015, Burkhard et al., 2014, Villamagna et al., 2013). By considering complexity and the vertical and horizontal hierarchical organization of ecosystems, we propose a revisited interpretation of the cascade framework as three-dimensional. More emphasis is attributed to the correct functioning of the complex system that generates individual ecosystem services and associated benefits for humans.

To further develop the concept of ecosystem services we propose that ecosystem services are not the benefits, but generate benefits as an output, often expressed in terms of biomass. A service implies that there is exchange of information and/or interaction. Goods are thus interpreted as material vehicles for ecosystem service enjoyment. We also propose that ecosystem functions are not services, but ecological processes that act at ecosystem level and generate flows of services. Functions should be maintained to ensure a sustainable flow of services. It is important to acknowledge that functions should be conceived with a more holistic and bio-centric approach compared to ecosystem services, which can be individually identified and assessed. We also call for greater attention toward ecosystem sciences for understanding long-term ecosystem integrity and ecosystem functioning, and thus the resilience of an ecosystem against human driven disturbances, in order to secure the vital ecosystem services and sustainable use of natural resources (Currie, 2011; Müller et al., 2010). The concepts of resilience science (resilience, adaptability and vulnerability) in relation to ecosystem services represent an important area for further studies (Brand, 2008, Müller et al., 2010). For such purposes, our clarification could be advantageous.

Adopting a more comprehensive view on the definitions in the cascade framework gives increased rigor to critical ecosystem services issues such as the techniques to map and assess services. For example, EU member states need consistent definitions and measurements for easy comparison of ecosystem services status, gain or loss across countries. Following the adoption of the analytical framework, including a conceptual model and two typologies, for the EU, Maes et al. (2016) presented a first test of the framework and an assessment of existing indicators to map (or quantify) ecosystem services at the national scale (see also Maes et al., 2014). These recent studies, as well as that by Egoh et al. (2012), show that national statistics present the best data options for mapping provisioning ecosystem services such as agricultural production. This approach, where benefits generated (biomass) are used as a proxy of the service, is highly simplified. The unit of assessment is the benefit and not the service. Choosing a proxy such as biomass as representative of a certain service is common practice, whereas a model that simulates the generation of the good/resource, which involves the interaction functioning, is often left out. We suggest that this approach is not fully consistent with the theoretical framework. However, it is acceptable in this case because biomass is conditioned by ecological structure and functioning.

We hope the insight into systems ecology provided in this article will offer some ground for reflection, fueling further advancement of the classification, conceptualization and operationalization of ecosystem services. Considering the growing interest in natural capital accounting,^8^ it is important to establish a consistent conceptual ground to highlight the difference between intermediate functioning within the ecosystem (function), final flows (services) and assets (benefits) and their respective degree of complexity. Given the need for economic valuation of ecosystem services, it is important to choose the appropriate valuation techniques which explicitly target the real object of valuation and thus avoid to consider the single, simple asset being equal to the more complex service that generates that asset.

## Figures and Tables

**Table 1:** Definitions and examples of ecosystem services terminology according to selected peer-reviewed literature.

Author & proposed application	Biophysical structure	Process	Function	Ecosystem services	Good	Benefit
Bateman et al. (2011)	e.g. animals, birds, plants and their connections, etc.	e.g. nutrient cycling	Primary ecological processes	Flow of services (outcome of structure and processes) provided by ecological assets in some assessment period.	Any object or construct which generates human wellbeing (physical and non).	The change in human well-being generated by a good (use-value and non). The same good can generate different values, depending on the context.
Boyd and Banzhaf (2007)	See definition for ‘process’	Biological, chemical, and physical interactions between ecosystem components. Functions and processes are not end-products; they are intermediate to the production of final ecosystem services.	See definition for ‘process’	The use of ecological asset over some time period.	Things directly enjoyed or consumed by households.	A benefit e.g. recreation, arises from the joint use of final ecosystem services and conventional goods and services.
Fisher et al. (2009)	See definition for ‘ecosystem services’	See definition for ‘ecosystem services’	See definition for ‘ecosystem services’	They are ecological in nature, in that aesthetic values, cultural contentment and recreation are not ecosystem services. Ecosystem services are ecological components, functions and/or processes, as long as there are human beneficiaries.	na	A benefit has an explicit impact on changes in human wellfare, like more food, better hiking, less flooding. For example, aesthethic values, cultural contentment and recreation are benefit and not just a function of the ecosystem, but include other inputs like human capital, built capital, etc.
Maes et al. (2016)	The architecture of an ecosystem as a result of the interaction between the abiotic, physical environment and the biotic communities, in particular vegetation	Any change or reaction which occurs within ecosystems, physical, chemical or biological. Ecosystem processes include decomposition, production, nutrient cycling, and fluxes of nutrients and energy	Subset of the interactions between biophysical structures, biodiversity and ecosystem processes that underpin the capacity of an ecosystem to provide ecosystem services	The direct and indirect contributions of ecosystems to human wellbeing (TEEB, 2010). The actually used service.	The concept 'ecosystem goods and services' is synonymous with ecosystem services.	Positive change in wellbeing from the fulfilment of needs and wants (TEEB, 2010)
Müller and Burkhard (2012)	Biophysical structures and processes (ecosystem properties) are linked in the cascade component of ecosystem functions. They are understood as the basic producers of ecosystem services.	See definition for ‘biophysical strucuture’	Ecological integrity	Direct and indirect contributions of ecosystem structures and functions	na	intended as social, economic and personal well-being
Mononen et al. (2016)	Biophysical structures that create the basis for functioning of the ecosystem. Spatial perspective.	na	Functioning of ecosystem that is needed to produce ecosystem services. Temporal perspective.	na	The used share of the potential of ecosystem services. Benefts can be also non-material.	Economic, social, health (physical or spiritual) and intrinsic value of the benefit.
Spanenberg et al. (2014)	Biophysical structure *or* process includes habitat type	See definition for ‘biophysical strucuture’	e.g. wood production	Collecting or harvesting wood (that is the human activity of withdrawing the natural asset)	Contribution to aspects of well-being such as health and safety	Willingness to pay for more woodland or harvestable products.
TEEB (2010)	Biophysical structure or process = vegetation cover or Net Primary Productivity	see Biophysical structure	The potential that ecosystems have to deliver a service which in turn depends on ecological structure and processes.	Conceptualizations of the “useful things” ecosystems “do” for people, directly *and* indirectly	na	Welfare gains generated by ecosystem services
Wallace (2007)	na	The complex interactions (events, recreations or operations) among biotic and abiotic elements of ecosystems that lead to a definite result.	See definition for ‘process’	Benefits that people obtain from ecosystems; the outcomes sought through ecosystem management.	na	Preferred end-states of existence, including those required for human survival and reproductive success, which taken together circumscribe human well-being. These exclude intrinsic value.

**Table 2:** Proposed definitions of the cascade framework terminology.

Term	Definition	Examples^a^
Biophysical structure^b^	The setting for ecosystem components (biotic and abiotic). This also relates to the ecological pattern	Forest tree cover Inland water bodies
Process or function	An ecological interaction among components in an ecosystem over time. Processes may generate several ecosystem services.	Net primary production Carbon cycling Nutrient cycling
Ecosystem service	A flow generated by the ecosystem including ecological interactions and information which are useful to human beings. We therefore propose that ecosystem services do not include ecosystem components or goods, i.e. countable as (bio)mass unit. In addition, ecosystem services sometimes require human input, which does not necessarily mean human-made constructs like labour, industrial processing, benches or fishing roads.^a^	Generation of material from plants Carbon sequestration Water purification Aesthetic beauty of landscape
Good	Countable as a (bio)mass unit, it is a vehicle for ecosystem service enjoyment.	Wood biomass Amount of CO_2_ retained from the atmosphere Amount of pollutants retained from water bodies People enjoying outdoor recreation activities
Benefit	What is generated by the service and leads to a change in human well-being.	Availability of wood for multiple uses Healthier air to breath/climate change mitigation Availability of cleaner water (instead of water polluted by economic activities)

a Example of human input includes existence of a human being with his/her sensory and perceptional experiences.

b Existing literature often uses the term ecological structure as a synonym for biophysical structure. We however prefer the later term, because it also includes non-vegetated structures, such as dunes, aquifers or Rocky Mountains.

**Table 3:** Classification of ecosystem services (CICES) including the nature of ecosystem services, the cascade framework step, the Systems Ecology category, the most logic/common assessment technique and their degree of complexity.

	List of ecosystem services according to CICES	Cascade framework step	Systems Ecology category	Assessment technique
Provisioning	Cultivated crops	Benefit	Biomass	Statistical datasets
	Wild plants, algae and their outputs	Benefit	Biomass	Statistical datasets
	Wild animals and their outputs	Benefit	Biomass	Statistical datasets
	Plants and algae from in-situ aquaculture	Benefit	Biomass	Statistical datasets
	Animals from in-situ aquaculture	Benefit	Biomass	Statistical datasets
	Materials from plants, algae and animals for agricultural use	Benefit	Biomass	Statistical datasets
	Genetic materials from all biota	Benefit	Biomass/ information	Statistical datasets
	Reared animals and their outputs	Benefit	Biomass	Statistical datasets
	Surface water for drinking	Benefit	Biomass	Statistical datasets
	Ground water for drinking	Benefit	Biomass	Statistical datasets
	Fibers and other materials from plants, algae and animals for direct use or processing	Benefit	Biomass	Statistical datasets
	Surface water for non-drinking purposes	Benefit	Mass	Mainly statistical datasets
	Ground water for non-drinking purposes	Benefit	Mass	Mainly statistical datasets
	Plant-based resources	Benefit	Biomass	Statistical datasets
	Animal-based resources	Benefit	Biomass	Mainly statistical datasets
	Animal-based energy	Benefit	Biomass	Mainly statistical datasets
Regulating and maintenance	Bio-remediation by micro-organisms, algae, plants, and animals	Service	Interaction	Biophysical models and/or measures
	Filtration/sequestration/storage/accumulation by micro-organisms, algae, plants, and animals	Service	Interaction	Biophysical models and/or measures
	Filtration/sequestration/storage/accumulation by ecosystems	Service	Interaction	Biophysical models and/or measures
	Mediation of smell/noise/visual impacts	Service	Interaction	Biophysical models and/or measures
	Dilution by atmosphere, freshwater and marine ecosystems	Function		
	Hydrological cycle	Function		
	Water flow maintenance	Service	Interaction	Biophysical models
	Mass stabilization and control of erosion rates	Service	Interaction	Biophysical models
	Global climate regulation by reduction of greenhouse gas concentrations	Service	Interaction	Biophysical models
	Micro and regional climate regulation	Service	Interaction	Biophysical models
	Buffering and attenuation of mass flows	Service	Interaction	Biophysical models and/or measures; Geospatial models
	Flood protection	Service	Interaction	Biophysical models and/or measures; Geospatial models
	Storm protection	Service	Interaction	Biophysical models and/or measures; Geospatial models
	Pollination and seed dispersal	Service	Interaction	Biophysical models and/or measures; Geospatial models
	Maintaining nursery populations and habitats	Service	Interaction	Biophysical models and/or measures; Complex indicators integrated with geospatial models
	Pest and disease control	Service	Interaction	Biophysical models and/or measures; Geospatial models
	Ventilation and transpiration	Function		
	Weathering processes	Function		
	Decomposition and fixing processes	Function		
	Chemical condition of freshwaters	Biophysical structure		
	Chemical condition of salt waters	Biophysical structure		
Cultural	Experiential use of plants, animals and land-/seascapes in different environmental settings	Service	Information	Geospatial models/complex indicators
	Physical use of land-/seascapes in different environmental settings	Service	Information	Geospatial models/complex indicators
	Aesthetic	Service	Information	Geospatial models/complex indicators
	Education	Service	Information	Complex indicators
	Heritage, cultural	Service	Information	Complex indicators
	Entertainment	Service	Information	Complex indicators
	Scientific	Service	Information	Complex indicators
	Symbolic	Service	Information	Complex indicators
	Sacred and/or religious	Service	Information	Complex indicators
	Existence	Value		
	Bequest	Value		

The attempt is to develop the same examples throughout the ‘terminology chain’ to show that they are indeed different stage of the same process. E.g. to differentiate the carbon cycling as function from carbon sequestration as service from CO_2_ tons will (if ever) be the task of the biophysical model, i.e. only one of those stages will be mapped and assessed, it will depend on the technique used to assess (model or indicator or statistics).

## Footnotes

1 e.g. RUBICODE (Rationalizing Biodiversity Conservation in Dynamic Ecosystems), SCALES (Securing the Conservation of biodiversity across Administrative Levels and spatial, temporal, and Ecological Scales), OpenNESS (Operationalization of Natural Capital and Ecosystem Services) and ESMERALDA (Enhancing ecoSysteM sERvices mApping for poLicy and Decision mAking)

2 Note that some authors, e.g. Mononen et al. (2016) have suggested to highlight the process-like nature of ecosystem services delivery as socio-ecological systems, thus maintaining the holistic approach on the focus.

3 The cascade model does indeed include, after ‘benefit’, also the ‘value’ step that assigns to benefits a quantification in monetary terms. The economic valuation of ecosystem services is a field of research and applications that does not affect the specific conceptual analysis proposed in this paper. In order to keep focused on the main objectives of the paper, we thus choose not to include the ‘value’ box at this stage.

4 Note that genetic information is stored in DNA and transmitted across generations.

5 For example: at cell level on the one hand the integrated cell processes determine the functionality of the organs, on the other hand organs control the final biochemical results of cells; at the level of populations on the one hand the individuals and their interactions determine the properties of the populations, and on the other hand population determines the living framework for the individuals.

6 A malfunction of one level can be eliminated by replacing a few components on the lower level. e.g cells, organs and species can be replaced to better fit the new emergent conditions. Thus, the higher the level is, the less vulnerable it becomes.

7 This is the reason why in Table 3 what corresponds to ‘Biophysical structure’ and ‘Function’ is not classified in terms of Systems Ecology category, and Assessment technique are thus reported as grey cells.

8 Ref. http://unstats.un.org/unsd/envaccounting/eea_project/default.asp, http://ec.europa.eu/environment/nature/capital_accounting/index_en.htm, http://www.naturalcapitalcommittee.org/, and http://www.wavespartnership.org/en/natural-capital-accounting.

## References

[bib0005] Albert C., Galler C., Hermes J., Neuendorf F., von Haaren C., Lovett A. (2015). Applying ecosystem services indicators in landscape planning and management: the ES-in-Planning framework. Ecol. Indic..

[bib0010] Bateman I.J., Mace G.M., Fezzi C., Atkinson G., Turner K. (2011). Economic analysis for ecosystem service assessments. Environ. Resour. Econ..

[bib0015] Bennett E.M., Cramer W., Begossi A., Cundill G., Díaz S., Egoh B.N., Geijzendorffer I.R., Krug C.B., Lavorel S., Lazos E., Lebel L., Martín-López B., Meyfroidt P., Mooney H.A., Nel J.L., Pascual U., Payet K., Harguindeguy N.P., Peterson G.D., Prieur-Richard A.H., Reyers B., Roebeling P., Seppelt R., Solan M., Tschakert P., Tscharntke T., Turner B.L., Verburg P.H., Viglizzo E.F., White P.C.L., Woodward G. (2015). Linking biodiversity, ecosystem services, and human well-being: three challenges for designing research for sustainability. Curr. Opin. Environ. Sustain..

[bib0020] Boerema A., Rebelo A.J., Bodi M.B., Esler K.J., Meire P. (2016). Are ecosystem services adequately quantified?. J. Appl. Ecol..

[bib0025] Boyd J., Banzhaf S. (2007). What are ecosystem services? The need for standardized environmental accounting units. Ecol. Econ..

[bib0030] Braat L.C., de Groot R. (2012). The ecosystem services agenda: bridging the worlds of natural science and economics conservation and development, and public and private policy. Ecosyst. Serv..

[bib0035] Brand F. (2008). Critical natural capital revisited: ecological resilience and sustainable development. Ecol. Econ..

[bib0040] Burkhard B., Kandziora M., Hou Y., Müller F. (2014). Ecosystem service potentials, flows and demands-concepts for spatial localisation, indication and quantification. Landsc.

[bib0045] Convention on Biological Diversity, 1992. United Nations. https://www.cbd.int/doc/legal/cbd-en.pdf.

[bib0050] Convention on Biological Diversity, 1998. Report on the working of ecosystem approach. UNEP conference of the parties to the Convention on Biological Diversity.

[bib0055] Convention on Biological Diversity, 2010. Convention on Biological Diversity. COP Decision X/2.

[bib0060] Costanza R., Kubiszewski I. (2012). The authorship structure of ecosystem services as a transdisciplinary field of scholarship. Ecosyst. Serv..

[bib0065] Currie W.S. (2011). Tansley review Units of nature or processes across scales? The ecosystem concept at age 75. N. Phytol..

[bib0070] Dawkins R. (1982). The Extended Phenotype.

[bib0075] de Groot Rudolf S., Wilson Matthew A., Boumans Roelof M.J. (2002). A typology for the classification, description and valuation of ecosystem functions, goods and services. Ecol. Econ..

[bib0080] Dusenbery, B., 1992. Sensory Ecology: How Organisms Acquire and Respond to Information

[bib0085] Edson M.M., Foin T.C., Knapp C.M. (1981). Emergent properties and ecological research. Am. Nat..

[bib0090] Egoh B., Drakou E.G., Dunbar M.B., Maes J., Willemen L. (2012). Indicators for Mapping Ecosystem Services: A Review.

[bib0095] Faith D.P., Magallón S., Hendry A.P., Conti E., Yahara T., Donoghue M.J. (2010). Evosystem services: an evolutionary perspective on the links between biodiversity and human well-being. Curr. Opin. Environ. Sustain..

[bib0100] Fisher B., Turner R.K. (2008). Ecosystem services: classification for valuation. Biol. Conserv..

[bib0105] Fisher B., Turner R.K., Morling P. (2009). Defining and classifying ecosystem services for decision making. Ecol. Econ..

[bib0110] Guerry A.D., Polasky S., Lubchenco J., Chaplin-Kramer R., Daily G.C., Griffin R., Ruckelshaus M., Bateman I.J., Duraiappah A., Elmqvist T., Feldman M.W., Folke C., Hoekstra J., Kareiva P.M., Keeler B.L., Li S., McKenzie E., Ouyang Z., Reyers B., Ricketts T.H., Rockström J., Tallis H., Vira B. (2015). Natural capital and ecosystem services informing decisions: from promise to practice. PNAS.

[bib0115] Guilford T., Dawkins M.S. (1991). Receiver psychology and the evolution of animal signals. Anim. Behav..

[bib0120] Haines-Young R., Potschin M. (2009). Methodologies for Defining and Assessing Ecosystem Services. Final Report.

[bib0125] Haines-Young R., Potschin M., Raffaelli D., Frid C. (2010). The links between biodiversity, ecosystem services and human well-being. Ecosystem Ecology: A New Synthesis, BES Ecological Reviews Series.

[bib0130] Heink U., Hauck J., Jax K., Sukopp U. (2015). Requirements for the selection of ecosystem service indicators—the case of MAES indicators. Ecol. Indic..

[bib0135] Jørgensen S. (2012). Introduction to Systems Ecology.

[bib0140] Jax K. (2005). Function and functioning in ecology: what does it mean?. Oikos.

[bib0145] Kremen C. (2005). Managing ecosystem services: what do we need to know about their ecology?. Ecol. Lett..

[bib0150] Landers, D.H., Nahlik, A.M., 2013. Final ecosystem goods and services classification system (FEGS-CS), in Anonymous EPA United States Environmental Protection Agency. Report Number EPA/600/R-13/ORD-004914.

[bib0155] Liquete C., Cid N., Lanzanova D., Grizzetti B., Reynaud A. (2016). Perspectives on the link between ecosystem services and biodiversity: the assessment of the nursery function. Ecol. Indic..

[bib0160] Liquete C., Piroddi C., Macías D., Druon J.-N., Zulian G. (2016). Ecosystem services sustainability in the Mediterranean Sea: assessment of status and trends using multiple modelling approaches. Sci. Rep..

[bib0165] Müller F., Burkhard B. (2012). The indicator side of ecosystem services. Ecosyst. Serv..

[bib0170] Müller F., Burkhard B., Kroll F., Otto J.-C., Dikau R. (2010). Resilience, integrity and ecosystem dynamics: bridging ecosystem theory and management. Landform—Structure, Evolution, Process Control, Lecture Notes in Earth Sciences 115.

[bib0175] MA (2005). Millennium Ecosystem Assessment.

[bib0180] Maes J., Teller A., Erhard M., Murphy P., Paracchini M., Barredo J., Grizzetti B., Cardoso A., Somma F., Petersen J. (2014). Mapping and Assessment of Ecosystems and Their Services—Indicators for Ecosystem Assessments Under Action 5 of the EU Biodiversity Strategy to 2020.

[bib0185] Maes J., Liquete C., Teller A., Erhard M., Paracchini M.L., Barredo J.I., Grizzetti B., Cardoso A., Somma F., Petersen J. (2016). An indicator framework for assessing ecosystem services in support of the EU Biodiversity Strategy to 2020. Ecosyst. Serv..

[bib0190] Matthies B., D’Amato D., Berghäll S., Ekholm T., Hoen H., Holopainen J., Korhonen J., Lähtinen J., Mattila O., Toppinen A., Valsta L., Wang L., Yousefpour R. (2016). An Ecosystem Service-Dominant Logic? Integrating the ecosystem service approach and the service-dominant logic. J. Clean. Prod..

[bib0195] Mononen L., Auvinen A.P., Ahokumpu A.L., Rönka M., Aarras N., Tolvanen G., Kamppinen M., Viirret E., Kumpula T., Vihervaara P. (2016). National ecosystem service indicators: measures of social-ecological sustainability. Ecol. Indic..

[bib0200] Odum E.P. (1977). The emergence of ecology as a new integrative discipline. Science.

[bib0205] Oxford Dictionary online, 2014. www.oxforddictionaries.com.

[bib0210] Palmer M.A., Febria C.M. (2012). The heartbeat of ecosystems. Science (80-).

[bib0215] Parrish J.K., Edelstein-Keshet L. (1999). Complexity, pattern, and evolutionary trade-offs in animal aggregation. Science.

[bib0220] Potschin M., Haines-Young R., Potschin M., Haines-Young R., Fish R., Turner R.K. (2016). Defining and measuring ecosystem services. Routledge Handbook of Ecosystem Services.

[bib0225] Potschin M., Haines-Young R., Fish R., Turner R.K. (2016). Routledge Handbook of Ecosystem Services.

[bib0230] Rhodes, C., 2015. National ecosystem services classification system. http://unstats.un.org/unsd/envaccounting/seeaRev/meeting2013/EG13-BG-13.pdf.

[bib0235] Seppelt R., Dormann C.F., Eppink F.V., Lautenbach S., Schmidt S. (2011). A quantitative review of ecosystem service studies: approaches, shortcomings and the road ahead. J. Appl. Ecol..

[bib0240] Spangenberg J.H., von Haaren C., Settele J. (2014). The ecosystem service cascade: Further developing the metaphor. Integrating societal processes to accommodate social processes and planning, and the case of bioenergy. Ecol. Econ..

[bib0245] Spangenberg J.H., Settele J. (2010). Precisely incorrect? Monetising the value of ecosystem services. Ecol. Compl..

[bib0250] Staub C., Ott W., Heusi F., Klingler G., Jenny A., Hacki M., Hauser A. (2011). Indicators for Ecosystem Goods and Services: Framework, Methodology and Recommendations for a Welfare-related Environmental Reporting Environmental Studies No. 1102.

[bib0255] TEEB, (2010). The Economics of Ecosystems and Biodiversity.

[bib0260] UK NEA, 2011. UK National Ecosystem Assessment: Technical Report. UNEP-WCMC.

[bib0265] Villamagna A.M., Angermeier P.L., Bennett E.M. (2013). Capacity, pressure, demand, and flow: a conceptual framework for analyzing ecosystem service provision and delivery. Ecol. Compl..

[bib0270] Wallace K.J. (2007). Classification of ecosystem services: problems and solutions. Biol. Cons..

[bib0275] Zulian G., Maes J., Paracchini M.L. (2013). Linking land cover data and crop yields for mapping and assessment of pollination services in europe. Land.

